# Hesperidin and its zinc(ii) complex enhance osteoblast differentiation and bone formation: *In vitro* and *in vivo* evaluations

**DOI:** 10.1515/biol-2022-1032

**Published:** 2025-06-17

**Authors:** Pan Li, Jing Wang, Huan Wang, Songchun Liu, Qibin Zhang

**Affiliations:** Department of Orthopaedics, Lequn Branch, The First Hospital of Jilin University, Changchun, Jilin, 130000, China; Department of Orthopedics, Ezhou Central Hospital, Ezhou, Hubei, 436000, China; Department of Clinical Nutrition, Ezhou Central Hospital, Ezhou, Hubei, 436000, China; Department of Spinal Surgery, Xinchang Hospital Affiliated to Wenzhou Medical University·Xinchang County People’s Hospital, Shaoxing, Zhejiang, 312500, China

**Keywords:** hesperidin, zinc(ii), microRNA, osteoblast, bone, zebrafish

## Abstract

This investigation explores the impact of hesperidin and its zinc(ii) complex on osteoblast differentiation and subsequent bone formation. The biocompatibility of synthesized complexes (0–20 μg/mL) was assessed *in vitro* using mouse mesenchymal stem cells, while *in vivo* toxicity was evaluated using a chick embryo model. Both hesperidin and its zinc(ii) complex were found to be non-toxic at a concentration of 10 μg/mL. Notably, these compounds significantly increased alkaline phosphatase activity and enhanced calcium deposition. Molecular analyses revealed upregulation of Runx2 and type 1 collagen mRNA expression, along with increased levels of osteonectin and osteocalcin proteins, while negative regulators of osteoblast differentiation (Smad7, Smurf1, HDAC7) were downregulated. A new aspect of this study is demonstrating that the zinc(ii) complex of hesperidin uniquely enhances osteogenic activity compared to hesperidin alone, highlighting its potential to improve bone formation significantly. Additionally, we elucidated the role of miR-143-3p in mediating these effects, achieved through HDAC7 suppression and enhanced Runx2 expression, assessed using the pmirGLO dual luciferase reporter system. Zebrafish studies further demonstrated the complexes’ effects on bone formation, revealing increased osteoblastic activity and improved calcium-to-phosphorus ratios in regenerated scales. These findings underscore the potential of hesperidin–Zn(ii) as a promising therapeutic agent for bone tissue engineering.

## 1 Introduction

Hesperidin, a natural bioflavonoid compound commonly found in citrus fruits such as oranges, lemons, tangerines, and grapefruits [1] possesses a wide range of biological properties, including anti-inflammatory, antioxidant, anti-aging, anticancer, and antibacterial effects [2,3,4]. Recent research indicates that flavonoid compounds, like hesperidin, play a crucial role in improving skeletal health and addressing conditions such as osteoporosis. These compounds act through various pathways to enhance bone density, such as by reducing oxidative stress and inflammation, thus promoting osteoblast formation and osteoclast differentiation [5,6,7]. Numerous studies have demonstrated hesperidin’s potential in preventing bone loss and facilitating bone defect regeneration. Additionally, the literature suggests that hesperidin may stimulate osteogenesis [6], which investigated its effects on preosteoblast cell function, osteogenesis, and collagen matrix quality. Their findings suggest that hesperidin regulates mineralized tissue formation by modulating osteoblast differentiation and controlling the ratio between matrix tissue and mineral.

Metal ions play indispensable roles in life’s metabolic processes. Among various compounds, flavonoids are recognized as efficient chelators of several metal ions, including zinc, iron, and copper. Flavonoid metal complexes have emerged as potential contributors to bone formation, presenting promising avenues for bone tissue regeneration. Research suggests that these complexes exhibit enhanced activity compared to their individual flavonoid counterparts. For instance, zinc/copper–silibinin complexes have demonstrated superior potential in promoting angiogenesis and osteogenesis relative to natural silibinin activity. Similarly, the quercetin–copper combination has shown enhanced angiogenesis and osteogenesis compared to native quercetin. Furthermore, the Kaempferol–zinc(ii) combination has been observed to enhance bone development in zebrafish, surpassing the stimulation achieved by Kaempferol alone [8]. Zinc (Zn), a prevalent trace element, forms complexes with flavonoids, displaying robust biological activities in cellular signaling pathways, particularly those involved in bone growth. Additionally, copper is a critical trace mineral vital for maintaining optimal bone health. Animal studies have demonstrated that a copper-deficient diet leads to reduced bone mineral content and strength [9]. Therefore, this study aims to synthesize a hesperidin–zinc(ii) complex to enhance the osteogenic activity of hesperidin. We hypothesize that this complex will improve the osteogenic properties of hesperidin, promoting increased osteoblast differentiation and mineralization. Moreover, it may exhibit superior effects on bone formation compared to hesperidin alone by modulating key cellular pathways and enhancing bioavailability.

## 2 Materials and methods

### 2.1 Synthesis of hesperidin–zinc(ii) complex

To synthesize the hesperidin–zinc(ii) complex, 1.00 × 10^−3^ mol/L of hesperidin (purchased from Sigma Aldrich) was mixed with an equal concentration of ZnCl_2_ in a ratio of 4:1 [10]. The resulting blended liquid, totaling 500 mL, was transferred into a round-bottom flask and subjected to heating, stirring, and refluxing at 70°C for 3 h until complete dissolution was achieved. Subsequently, the pH of the mixture was adjusted to 10.50 using 0.1 mol/L NaOH, and after allowing the solution to stand for 5 min, it was concentrated to 50 mL using a rotary evaporator. The residue obtained was washed multiple times with absolute methanol to eliminate impurities. Finally, the complex was dried under a vacuum for 12 h and utilized as a sample for infrared analysis.

### 2.2 Cell culture and MTT assay

For cell culture and MTT assay, immortalized mouse mesenchymal stem cells (MSCs), specifically C3H10T1/2, were acquired from NCCS, Pune, India, and maintained in DMEM supplemented with 10% FBS under standard conditions at 37°C with 5% CO_2_. The MTT assay was conducted following established protocols. Briefly, cells were seeded at a density of 2 × 10^4^ cells per well in 96-well plates and allowed to adhere for 12 h. Subsequently, the cells were treated with varying concentrations (ranging from 0 to 20 μg/mL) of either hesperidin or the hesperidin–zinc(ii) complex for up to 48 h. Following the incubation period, MTT solution at a concentration of 5 mg/mL was added to each well, and the plates were further incubated at 37°C for 4 h. Absorbance readings were taken at 570 nm. After treatment, the cells were washed and incubated with Fluorescein diacetate (FDA), a live cell staining solution at a concentration of 30 μg/mL for 5 min. The cellular morphology was then examined under a fluorescent microscope.

### 2.3 Alkaline phosphatase (ALP) activity assay

On day 14 of post-osteogenic induction, the assessment of ALP activity was conducted. ALP activity was determined utilizing an ALP activity kit following the manufacturer’s instructions provided by Beyotime. The absorbance of the samples was measured at 405 nm wavelength.

### 2.4 Alizarin red staining

After the treatment, the cell samples underwent a thorough washing procedure with phosphate-buffered saline (PBS), aimed at removing any extraneous debris or residual media. Subsequently, the cells were fixed by immersion in 95% ethanol for a duration of 10 min, ensuring the preservation of their structural integrity. Following fixation, the samples underwent another round of washing with PBS, which served to eliminate any excess ethanol and prepare the cells for staining. The cell cultures were then covered with a 0.1% solution of Alizarin red and allowed to incubate for 10 min, facilitating the specific staining of calcium deposits within the nodules. Upon completion of the incubation period, the samples were rinsed meticulously with PBS to remove any unbound dye molecules. Finally, the stained cell cultures were subjected to observation under an inverted light microscope to visualize and analyze the presence and distribution of calcified nodules.

### 2.5 Real-time RT-PCR analysis

Total RNA extraction was conducted utilizing TRIzol reagent (Invitrogen), following the manufacturer’s protocol to ensure efficient isolation. Subsequently, complementary DNA (cDNA) synthesis was performed using the RevertAid First Strand cDNA Synthesis Kit (K1622, Thermo Fermentas), employing the isolated RNA as the template. For quantitative PCR amplification, iTaq Universal SYBR Green Supermix (Bio-Rad) was utilized according to the manufacturer’s instructions. To ensure accuracy and reliability, GAPDH or U6 served as internal reference controls for normalization purposes. The primer sequence used is listed in Table S1 [11,12]. The gene expression levels were quantified using the 2^−ΔΔCt^ method, which allows for the comparative analysis of gene expression between different samples while considering variations in PCR efficiency and RNA input.

### 2.6 ELISA quantification of osteonectin (ON) and osteocalcin (OC)

Following treatment with hesperidin or hesperidin–zinc(ii) complex, the conditioned medium containing secreted proteins was collected from the cells. The levels of OC and ON were quantified using commercially available ELISA Kits (Elabscience) according to the manufacturer’s instructions. Briefly, the conditioned medium samples were added to the ELISA plate pre-coated with specific antibodies for OC and ON. After an appropriate incubation period allowing for binding of the target proteins, the unbound substances were washed away. Subsequently, enzyme-conjugated secondary antibodies were added to facilitate detection. Following another washing step to remove excess conjugates, a substrate solution was added to induce a colorimetric reaction. The intensity of the resulting color was measured spectrophotometrically at a specific wavelength corresponding to the detection of OC and ON. The concentrations of OC and ON in the samples were determined by comparing the absorbance values to standard curves generated using known concentrations of OC and ON provided with the ELISA Kits. This method allowed for accurate quantification of OC and ON levels in the conditioned medium, providing valuable insights into the effects of hesperidin or hesperidin–zinc(ii) complex on the secretion of these osteogenic markers.

### 2.7 Toxicity assessment of hesperidin and hesperidin–zinc(ii) complex on chick embryo development: a morphological evaluation

Fertilized leghorn chicken eggs procured from a local authorized dealer were utilized to investigate the toxicity of hesperidin or hesperidin–zinc(ii) complex following established protocols [13]. Injection of various concentrations of hesperidin or hesperidin–zinc(ii) complex was performed on chick embryos at the HH-12 stage (day 2) of development, with double-distilled water serving as the control. The experiment encompassed multiple replicates (*n* = 30) to ensure statistical robustness. Subsequent to treatment, embryos were dissected at the HH-37 stage (11th day) to assess morphological alterations, enabling a thorough evaluation of hesperidin and hesperidin–zinc(ii) complex toxicity on chick embryo development and yielding insights into their safety profiles.

### 2.8 Assessment of bone formation effects of hesperidin and hesperidin–zinc(ii) complex using the zebrafish scale model

The zebrafish (Danio rerio) scale model was employed to examine the effects of hesperidin and hesperidin–zinc(ii) complex on bone formation. Adult zebrafish, aged 3 months, were exposed to 10 μg/mL of hesperidin and hesperidin–zinc(ii) complex for a period of 14 days. Scales were carefully excised from each fish at day zero, totaling 30 scales per fish, and the fish were then maintained under the designated treatment conditions. After 14 days, the regenerating scales were collected for further analysis. The removed scales were fixed in 4% paraformaldehyde and subjected to von Kossa staining to assess calcium deposition, utilizing a procedure involving silver nitrate staining under ultraviolet light, which selectively stains calcium deposits black. Mineral analysis of the scales was performed using inductively coupled plasma mass spectrometry (ICP-MS). The scales were dissolved in 65% nitric acid and diluted 100 times with MilliQ water for subsequent ICP-MS analysis. Total phosphorus, calcium, and magnesium contents were quantified, and the calcium/phosphorus molar ratio was calculated to evaluate mineral composition. This integrated approach facilitated the comprehensive assessment of bone mineralization and composition in response to hesperidin and hesperidin–zinc(ii) complex in the zebrafish scale model.


**Ethical approval:** The research related to animal use has been complied with all the relevant national regulations and institutional policies for the care and use of animals and has been approved by the institutional animal ethics committee.

### 2.9 Assessment of microRNA (miRNA) regulation on HDAC7 3′UTR constructs using a luciferase reporter assay

The wild-type or mutant 3′UTR sequences of HDAC7 were chemically synthesized. The sequences were cloned between the PmeI and XbaI restriction sites in the pmirGLO dual-luciferase miRNA target expression vector (Promega), incorporating an internal NotI site. The resulting constructs were screened for the presence of the HDAC7 3′UTR region using the NotI restriction enzyme. MG63 cells were transiently transfected with either wild-type or mutant HDAC7 3′UTR constructs (200 ng) along with control miRNA or miR-143-3p mimic (50 nM) using Lipofectamine-2000 transfection reagent (Invitrogen). After 24 h, luciferase activity was measured using a dual-luciferase reporter assay kit (Promega). Firefly luciferase activity was normalized to Renilla luciferase activity according to established protocols [14]: 3′UTR of HDAC7 wild (sense 5′ AAAC TA GCGGCCGC TAGT ATGGTGCCAAACCCTTCATCTC T 3′ and antisense 3′ TTTG AT CGCCGGCG ATCA TACCACGGTTTGGGAAGTAGAG AGATC 5′) and 3′UTR of HDAC7 mutant (sense 5′ AAAC TA GCGGCCGC TAGT ATGGTGCCAAACCCTTCTATTC T 3′ and antisense 3′ TTTG AT CGCCGGCG ATCA TACCACGGTTTGGGAAGATAAG AGATC 5′).

### 2.10 Statistical analysis

All experimental results underwent validation through a minimum of three independent experiments, ensuring reliability. Statistical analyses were carried out employing SPSS 20.0 software to ascertain significance. The outcomes were presented as mean values accompanied by standard deviations to depict data dispersion accurately. Quantitative data were subjected to comparison utilizing one-way analysis of variance and Student’s *t*-test, facilitating the assessment of intergroup differences. A significance threshold of *p* < 0.05 was established for all statistical evaluations, ensuring robustness and reliability in the interpretation of results.

## 3 Results and discussion

### 3.1 Comprehensive evaluation of the biocompatibility of hesperidin and its zinc(ii) complex

**Figure 1:**
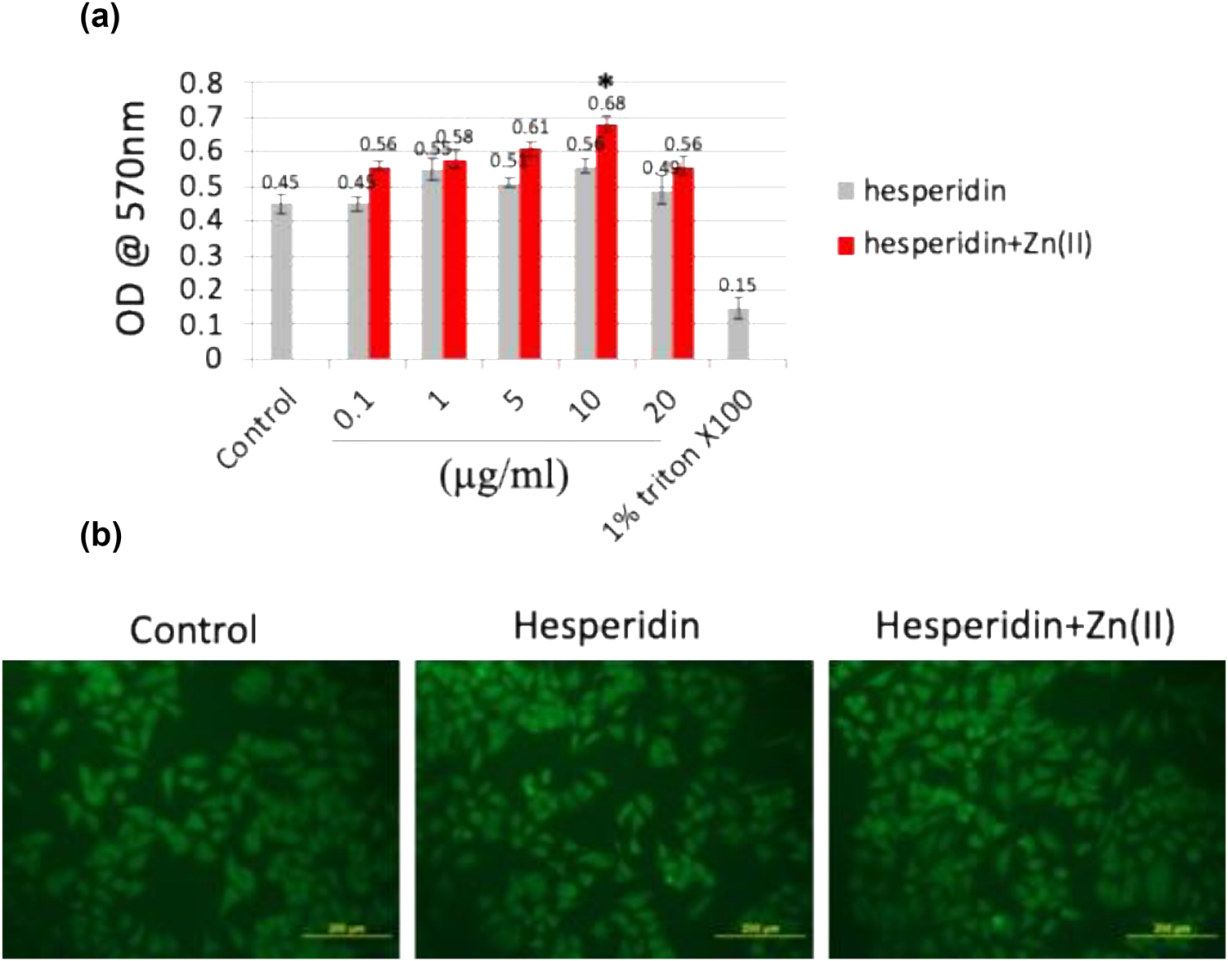
(a) The MTT assay was conducted to assess the cellular metabolic activity of mouse MSC cells exposed to various concentrations (0–20 μg/mL) of hesperidin and hesperidin–Zn(ii) complexes for up to 48 h. (b) FDA staining was performed to visualize cell morphology and viability after treatment with 10 μg/mL of hesperidin and hesperidin–Zn(ii) complexes for 24 h, followed by observation under a fluorescent microscope. Scale bar: 200 μm. * indicates a statistically significant increase compared to control.

The characterization of the hesperidin–zinc(ii) complex has been extensively reported in the previous manuscript. Various techniques were employed to study the structure of the complex, including scanning electron microscopy (SEM)–energy-dispersive X-ray spectroscopy for morphological analysis, X-ray diffraction for crystallographic information, Fourier-transform infrared spectroscopy for functional group identification, thermogravimetric analysis for thermal stability, and differential scanning calorimetry for phase transitions. These methods collectively provide a comprehensive understanding of the properties and behavior of the hesperidin–zinc(ii) complex [15,16,17,18]. The biocompatibility of hesperidin and its zinc(ii) complex was thoroughly evaluated using both *in vitro* and *in vivo* investigations, with the goal of understanding their effects on osteoblast development and bone formation. At first, mouse MSCs were tested using the MTT assay to measure their cellular reaction to different doses (ranging from 0 to 20 μg/mL) of hesperidin and hesperidin–Zn(ii) complex during a 48-h timeframe. Figure 1a demonstrates the metabolic activity of the cells after treatment, showing a significant increase in cellular activity, especially at a concentration of 10 μg/mL of the hesperidin–Zn(ii) complex. Surprisingly, there were no indications of toxicity seen at concentrations of up to 10 μg/mL for both hesperidin and its zinc(ii) complex. In addition, the impact of a concentration of 10 μg/mL of hesperidin and hesperidin–Zn(ii) complex on the shape and structure of osteoblasts was assessed by FDA staining, as shown in Figure 1b. The acquired pictures showed well-dispersed osteoblasts with distinct cytoplasmic extensions, indicating favorable circumstances for cell development. Furthermore, the biocompatibility of hesperidin and its complex was evaluated *in vivo* using a chick embryo model. Table 1 displays the examination of physical characteristics after treatment, indicating that there were no noticeable changes in chick embryo growth up to a concentration of 10 μg/mL for both substances. Moreover, zebrafish embryos that were subjected to hesperidin and its zinc(ii) complex at a concentration of up to 10 μg/mL did not display any harmful or negative effects (unpublished data). Based on the outcomes of biocompatibility tests conducted in a laboratory setting and on living organisms, it was determined that a concentration of 10 μg/mL for both hesperidin and its zinc(ii) complex is the most suitable for further investigations.

**Table 1:** The results of toxicity studies conducted on hesperidin and hesperidin–Zn complexes using a chick embryo model

Group	Animal condition	Heart rate (Bpm)	Weight of body (g)	Weight of heart (mg)	Length of animal (cm)	Twisted neck (%)	Hemorrhage (rupture blood vessels) (%)	Microphthalmia (abnormal eyes) (%)	Polydactyly (extra finger) (%)	Micromelia (smallness of one limb) (%)	Excencephaly (brain outside skull) (%)	Omphalocele (organ develop outside) (%)
Control	Live	56.6 ± 5.2	4.1 ± 1.4	62 ± 9	5.3 ± 1.3	0	0	0	0	0	0	0
Hesperidin (10 µM)	Live	56.6 ± 4.3	4.1 ± 1.2	61 ± 12	5.3 ± 1.2	0	0	0	0	0	0	0
Hesperidin (20 µM)	Live	56.7 ± 4.5	4.2 ± 1.3	61 ± 10	5.2 ± 1.2	0	0	0	0	0	0	0
Hesperidin–Zn(ii) (10 µM)	Live	56.2 ± 3.4	4.2 ± 1.4	61 ± 12	5.2 ± 1.1	0	0	0	0	0	0	0
Hesperidin–Zn(ii) (20 µM)	Live	56.3 ± 3.3	4.3 ± 1.5	61 ± 11	5.4 ± 1.1	0	0	0	0	0	0	0

The percentage mean values for each group were obtained from three independent experiments, with the SEM indicated as ±. Each treatment group consisted of 30 eggs.

### 3.2 Hesperidin and its zinc(ii) complex enhance the differentiation of mouse MSCs into osteoblasts

**Figure 2:**
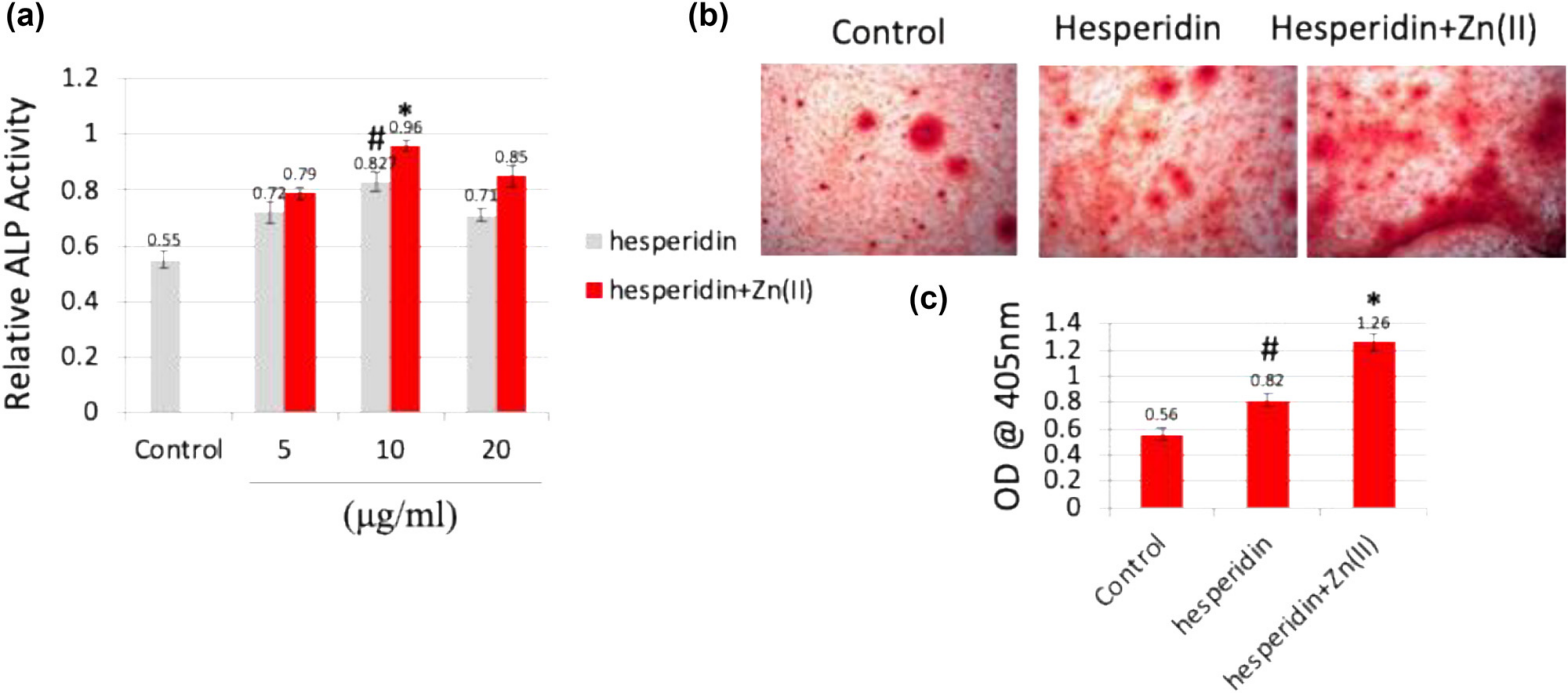
Hesperidin and hesperidin–Zn(ii) complex promoted mouse MSC differentiation toward osteoblasts at the cellular level. (a) Mouse MSCs were treated with different concentrations (5–20 μg/mL) of hesperidin and hesperidin–Zn(ii) complexes for up to 7 days, and ALP activity was measured. (b) Cells treated with 10 μg/mL of hesperidin and hesperidin–Zn(ii) complexes for 7 days were subjected to Alizarin red staining to assess calcium deposition. (c) Quantification of calcium deposition. * indicates a significant increase compared to the control, while # indicates a significant increase compared to hesperidin.

**Figure 3:**
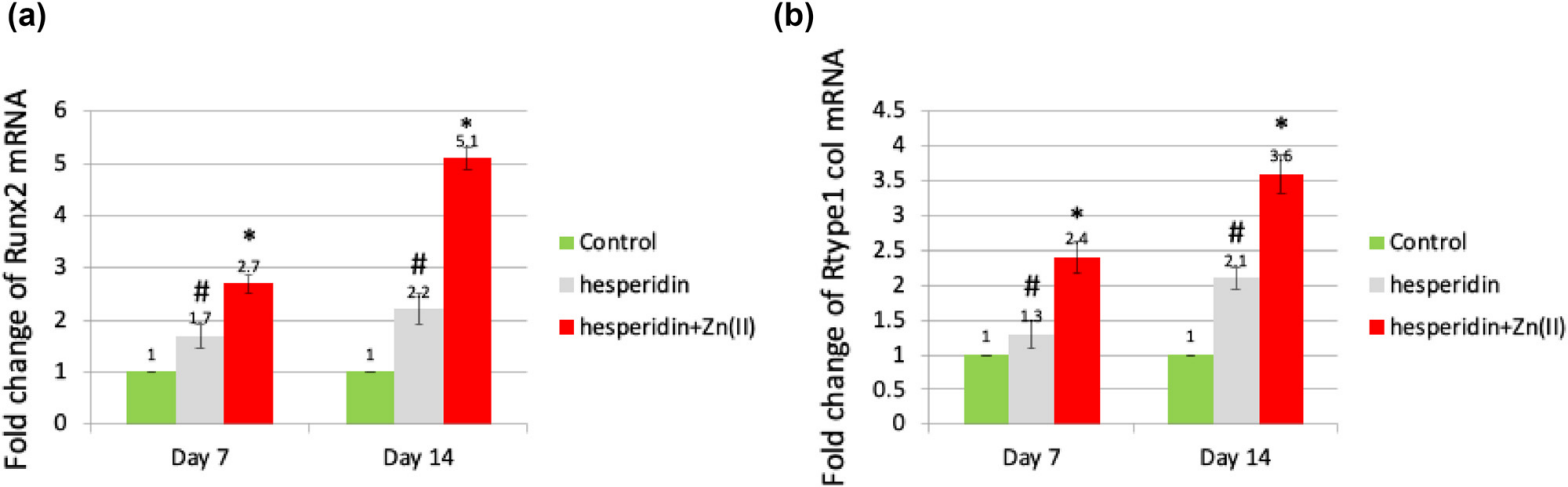
Hesperidin and hesperidin–Zn(ii) complexes increased osteoblast marker gene expression in mouse MSCs. Mouse MSCs were treated with 10 μg/mL of hesperidin and hesperidin–Zn(ii) complexes for up to 14 days. Total RNA was isolated, and the expression of Runx2 mRNA (a) and type 1 collagen mRNA (b) was analyzed by real-time RT-PCR. * indicates a significant increase compared to the control, while # indicates a significant increase compared to hesperidin.

Exposing mouse MSCs to varying concentrations (0–20 μg/mL) of hesperidin and hesperidin–zinc(ii) complex enabled the assessment of osteoblast differentiation at both cellular and molecular levels. Initially, after 7 days of treatment, ALP activity was measured (Figure 2a), where 10 μg/mL of both hesperidin and hesperidin–Zn(ii) complex exhibited notably higher activity compared to other concentrations. ALP, produced by osteoblasts, plays a crucial role in bone matrix calcification and serves as a precursor marker for osteoblastic cells, under the influence of Runx2, a critical bone transcription factor [8]. Remarkably elevated ALP activity was observed at 10 μg/mL concentration of both hesperidin and hesperidin–Zn(ii) complex. Furthermore, to elucidate the role of these compounds in calcium deposition, mouse MSCs were treated with 10 μg/mL hesperidin and the hesperidin–Zn(ii) complex for up to 14 days (Figure 2b and c). Post-treatment, cells were stained with Alizarin red (Figure 3b), and calcium deposition was quantified (Figure 2c). Results revealed that hesperidin increased calcium deposition compared to the control, with further enhancement observed with the hesperidin–Zn(ii) complex. The cellular-level effects of hesperidin and hesperidin–Zn(ii) complex positively regulated osteoblast differentiation, with the 10 μg/mL concentration deemed optimal for osteoblastic stimulation.

To clarify the effects of hesperidin and its zinc(ii) complex on the process of osteoblast differentiation, we exposed mouse MSCs to different concentrations (ranging from 0 to 20 μM) of hesperidin and hesperidin–Zn(ii) complex. We then assessed the levels of osteoblast differentiation at both the cellular and molecular levels. After 7 days of treatment, the activity of ALP was evaluated (Figure 2a). Both hesperidin and its zinc(ii) complex, when present at a concentration of 10 μg/mL, showed a notably increased ALP activity compared to other concentrations. ALP is an essential signal produced by osteoblasts that play a critical role in the calcification of the bone matrix. Its regulation is controlled by Runx2, a key transcription factor involved in bone development [8]. The significant rise in ALP activity observed at a concentration of 10 μg/mL of hesperidin and hesperidin–Zn(ii) combination indicates their strong ability to stimulate osteoblasts.

To investigate the impact of hesperidin and its complex on calcium deposition, mouse MSCs were exposed to a concentration of 10 μg/mL hesperidin and the hesperidin–Zn(ii) combination for a duration of 14 days (Figure 2b and c). After the treatment, Alizarin red staining was performed to observe the deposition of calcium (Figure 3b), and then the amount of calcium deposition was measured (Figure 2c). The findings demonstrated that the administration of hesperidin alone resulted in an increase in calcium deposition compared to the control group. Additionally, the hesperidin–Zn(ii) combination further enhanced this effect.

In summary, the impact of hesperidin and its zinc(ii) complex on osteoblast development is clearly demonstrated at the cellular level, highlighting their ability to enhance this process. The most effective concentration for promoting osteoblastic activity seems to be 10 μg/mL for both hesperidin and hesperidin–Zn(ii) combination. The results emphasize the potential of hesperidin and its zinc(ii) complex as effective agents for enhancing the differentiation of osteoblasts and the creation of bone. This suggests that more research should be conducted to explore their potential applications in bone tissue engineering.

To investigate the molecular processes involved in osteoblast differentiation, we treated mouse MSCs with 10 μg/mL concentrations of hesperidin and hesperidin–Zn(ii) complex for a duration of 14 days. Subsequently, we evaluated the expression of genes that serve as markers for osteoblasts. Runx2, a crucial controller, coordinates the process of osteoblast development by stimulating the production of certain indicators such as ALP, type 1 collagen (Col1), OC, and ON [19].

At first, the levels of Runx2 expression (Figure 3a) and type 1 collagen mRNA (Figure 3b) were evaluated. The findings demonstrated that administering 10 μg/mL hesperidin notably increased the expression of Runx2 and type 1 collagen mRNAs on both day 7 and day 14, in comparison to the control group. In addition, the levels of expression were increased even more by the treatment with the 10 μg/mL hesperidin–Zn(ii) complex, compared to both the control and hesperidin alone.

**Figure 4:**
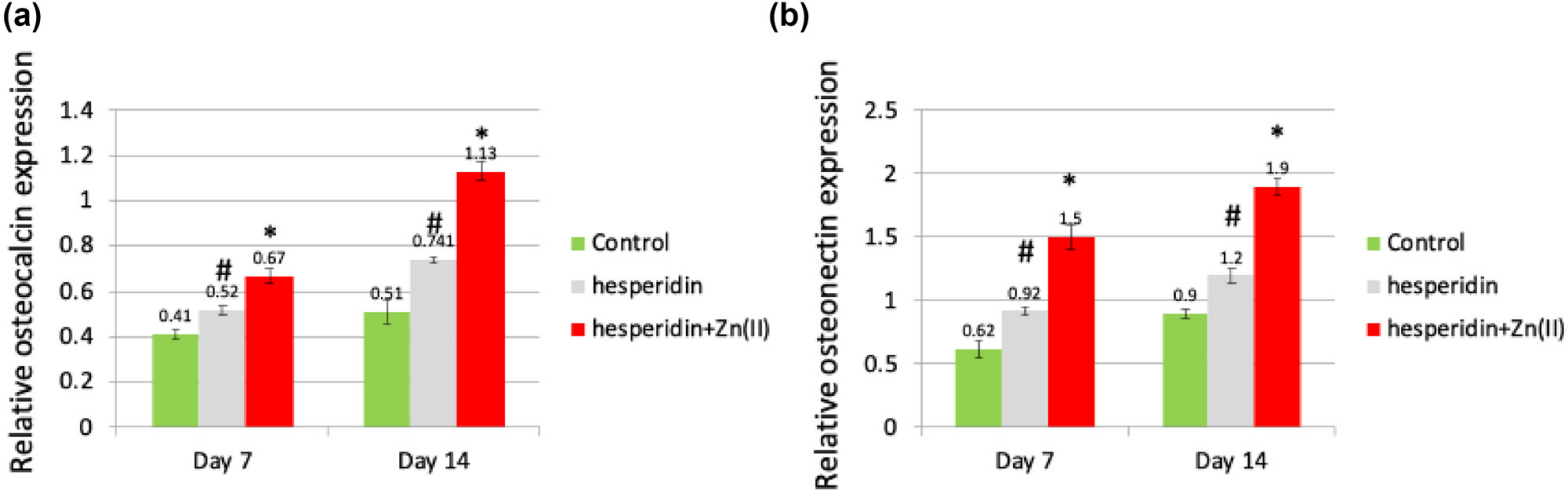
Hesperidin and hesperidin–Zn(ii) complexes increased OC and ON secretion levels in mouse MSCs during osteoblast differentiation. Mouse MSCs were treated with 10 μg/mL of hesperidin and hesperidin–Zn(ii) complexes for up to 14 days. The levels of OC and ON in the conditioned medium were measured. * indicates a significant increase compared to the control, while # indicates a significant increase compared to hesperidin.

In addition, ALP activity was quantified on day 7 (Figure 2a), while the secretion levels of OC and ON were evaluated on both day 7 and day 14 after treatment with 10 μg/mL hesperidin and hesperidin–Zn(ii) complex (Figure 4). The results demonstrated that hesperidin significantly increased ALP activity, OC, and ON levels compared to the control group at both time points, which is consistent with the expression patterns of osteoblast marker genes Runx2 and type 1 collagen. Furthermore, the administration of the hesperidin–Zn(ii) complex enhanced these effects much more when compared to hesperidin alone. The molecular study showed that 10 μg/mL hesperidin significantly increased the expression of Runx2 and type 1 collagen mRNAs, as well as ALP activity, OC, and ON secretion at both day 7 and day 14 compared to the control group. Significantly, the presence of zinc(ii) in hesperidin increased the observed effects, indicating a synergistic improvement in osteoblast differentiation when hesperidin is combined with zinc(ii) compared to hesperidin alone.

### 3.3 Modulation of Runx2 co-repressors by hesperidin and its zinc(ii) complex in osteoblast differentiation

**Figure 5:**
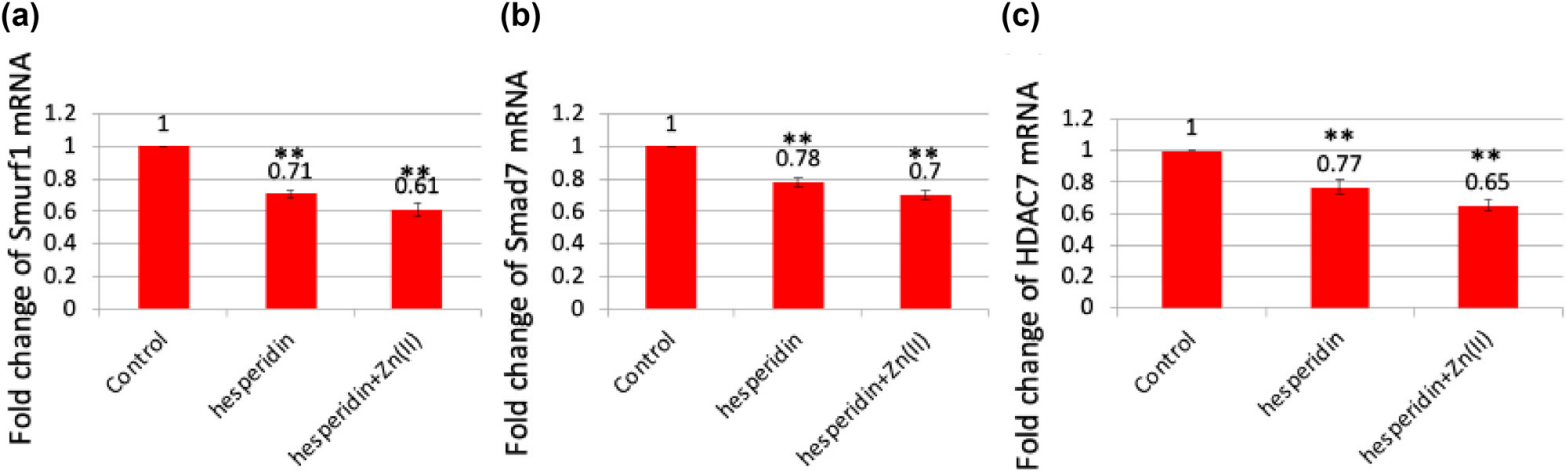
Hesperidin and hesperidin–Zn(ii) complexes reduced the expression of negative regulator genes associated with osteoblast differentiation. Mouse MSCs were treated with 10 μg/mL of hesperidin and hesperidin–Zn(ii) complexes for up to 14 days. Total RNA was isolated, and real-time RT-PCR analysis was performed to measure the expression levels of smad7, Smurf1, and HDAC7 mRNAs. ** indicates a significant decrease compared to the control.

The results depicted in Figures 1–4 clearly establish the ability of hesperidin and its zinc(ii) complex to enhance the development of osteoblasts. Figure 2 illustrates that both hesperidin and hesperidin–Zn(ii) complex greatly enhanced the expression of Runx2, an essential transcription factor involved in the development of osteoblasts. Expanding on this result, we performed additional investigation on the levels of expression of Runx2 co-repressors, specifically Smad7, Smurf1, and HDAC7, as depicted in Figure 5. Smad7, a type of Smad that inhibits the activity of other Smads, has a negative effect on the signaling pathways of BMP and TGF-β. It does this by blocking the transmission of signals from regulatory Smads. As a result, the expression of Runx2 is reduced and bone formation is inhibited [14]. Smurf1, an E3 ubiquitin ligase with an HECT domain, plays a role in breaking down important proteins that are involved in the process of osteoblast differentiation. This includes proteins like Runx2 and BMP receptors. As a result, Smurf1 has a negative regulatory effect on this process. HDAC7, a histone deacetylase, has been linked to the suppression of osteoblast development by reducing the activity of Runx2 through many methods [20]. Our findings from Figure 5 demonstrate that both hesperidin and its zinc(ii) complex effectively reduced the expression of Smad7, Smurf1, and HDAC7 in comparison to the control. This suggests that they have the potential to alleviate the inhibitory effects on osteoblast differentiation caused by these regulatory proteins.

### 3.4 Regulation of osteoblast differentiation by hesperidin and its zinc(ii) complex via miR-143-3p/HDAC7 signaling pathway

**Figure 6:**
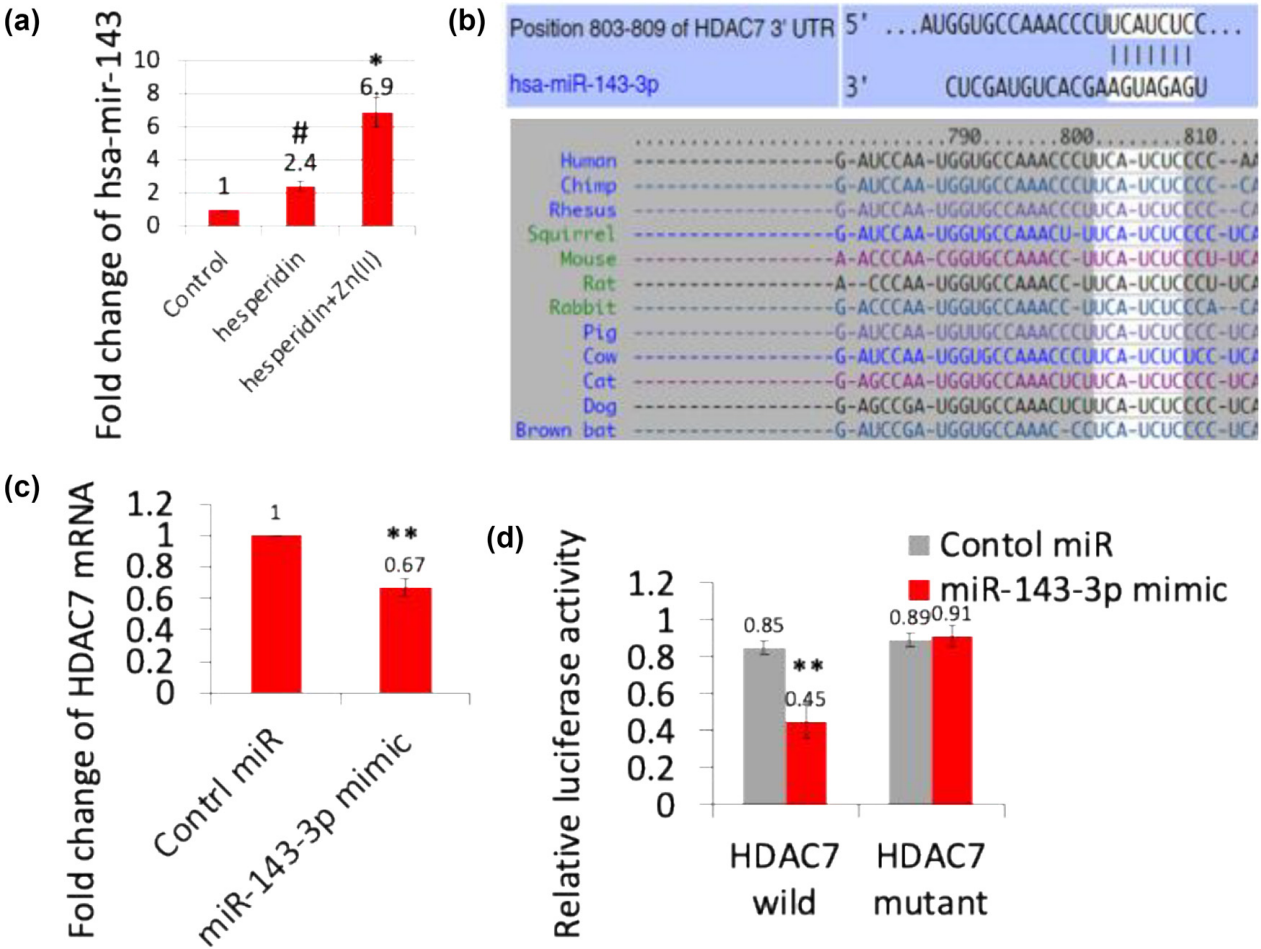
Hesperidin and hesperidin–Zn(ii) complexes modulate mir-143 expression in mouse MSCs. Mouse MSCs were treated with 10 µg/mL of hesperidin and hesperidin–Zn(ii) complexes for up to 14 days. (a) Total RNA was isolated, and mir-143 expression was analyzed by real-time RT-PCR. (b) HDAC7 was identified as the predicted target gene for miR-143-3p. (c) Transfection of MG63 cells with miR-143-3p mimic resulted in decreased HDAC7 mRNA and protein expression. (d) Luciferase reporter assay showing direct targeting of HDAC7 3′UTR by miR-143-3p. Wild-type or mutant HDAC7 3′UTR pmirGLO vectors were co-transfected with control miRNA or miR-143-3p mimic in MG63 cells, and luciferase activity was measured after 24 h. ** indicates a significant decrease compared to the control. */# indicates a significant increase compared to the control.

The role of miRNAs in controlling osteoblast differentiation through post-transcriptional regulation of gene expression is well-established. These small, non-coding RNA molecules have been shown to interact with the 3′UTR of specific mRNAs, resulting in their suppression [14]. Figure 5 clearly demonstrates that both hesperidin and its zinc(ii) complex significantly suppress the expression of HDAC7 during osteoblast differentiation. Recent literature suggests that miR-143 is expressed in osteoblasts and directly targets HDAC7 to promote osteoblast differentiation [20]. To investigate whether hesperidin and its zinc(ii) complex regulate miR-143 to control HDAC7 expression, mouse MSCs were treated with these compounds for up to 14 days, and total RNA was isolated to analyze precursor miR-143 expression via real-time RT-PCR (Figure 6a). As expected, the expression of miR-143 was increased by both hesperidin and its zinc(ii) complex. Furthermore, we predicted the putative target region of miR-143-3p within the 3’UTR of HDAC7 using TargetScan (http://www.targetscan.org/vert_72/) (Figure 6b). To confirm HDAC7 as a target of miR-143-3p, MG63 cells were transiently transfected with control miRNA or miR-143-3p mimic, followed by real-time RT-PCR analysis of HDAC7 expression (Figure 6c). Consistent with our hypothesis, the mRNA level of HDAC7 expression was decreased by miR-143-3p mimic compared to control miRNA transfection. To further confirm HDAC7 as a direct target of miR-143-3p, a dual-luciferase reporter assay was employed. Wild-type and mutant HDAC7 3′UTR sequences were synthesized and cloned into the pmirGLO vector. Co-transfection of wild-type HDAC7 3′UTR-containing pmirGLO vector with the miR-143-3p mimic resulted in significantly reduced luciferase activity compared to co-transfection with control miRNA (Figure 6d). However, co-transfection with mutant HDAC7 3′UTR-containing pmirGLO vector had no significant effect on luciferase activity. These findings provide experimental evidence that miR-143-3p directly targets the 3′UTR of the HDAC7 gene, highlighting the regulatory role of hesperidin and its zinc(ii) complex in osteoblast differentiation through the activation of the miR-143-3p/HDAC7 signaling pathway. The miRNA-143 specifically targeted the HDAC7 inhibitor, and reducing the expression of HDAC7 was seen to restore the function impaired by the deficit of miRNA-143. Therefore, miR-143 enhances the process of angiogenesis and osteoblast development by specifically targeting HDAC7. This suggests that HDAC7 could be a promising target for treating illnesses related to both angiogenesis and osteogenesis [20].

### 3.5 Enhancement of osteoblast differentiation by hesperidin and its zinc(ii) complex in a zebrafish scale model

**Figure 7:**
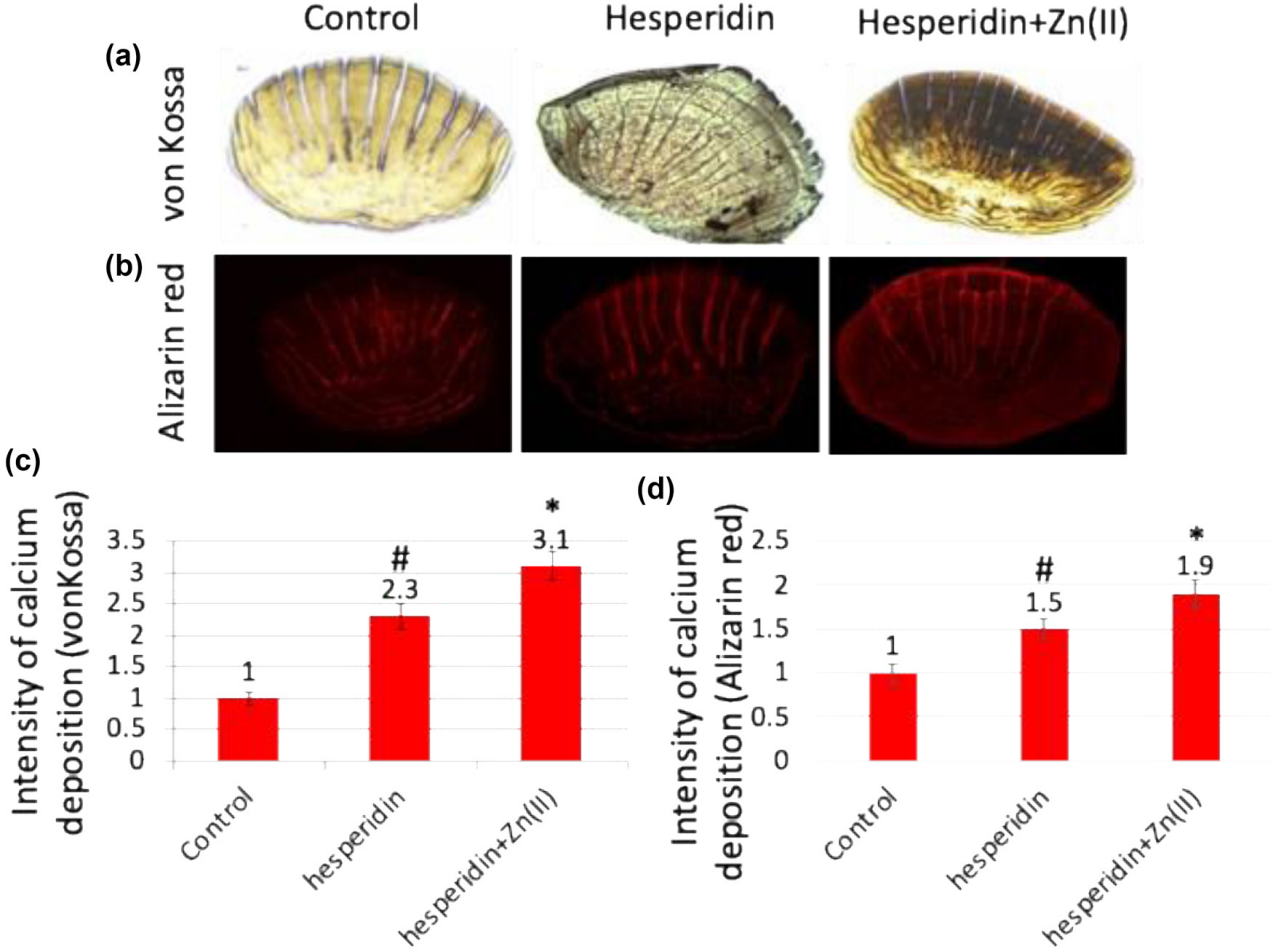
Hesperidin and hesperidin–Zn(ii) complexes enhance bone formation in the zebrafish scale model. Adult zebrafish were treated with 10 µg/mL of hesperidin and hesperidin–Zn(ii) complexes for up to 14 days. (a) Scales were stained with von Kossa staining to visualize calcium deposition. (b) Alizarin red staining was performed to assess mineralization. Quantification of calcium deposition was carried out based on von Kossa staining (c) and Alizarin red staining (d). * indicates a significant increase compared to the control, while # indicates a significant increase compared to hesperidin.

**Figure 8:**
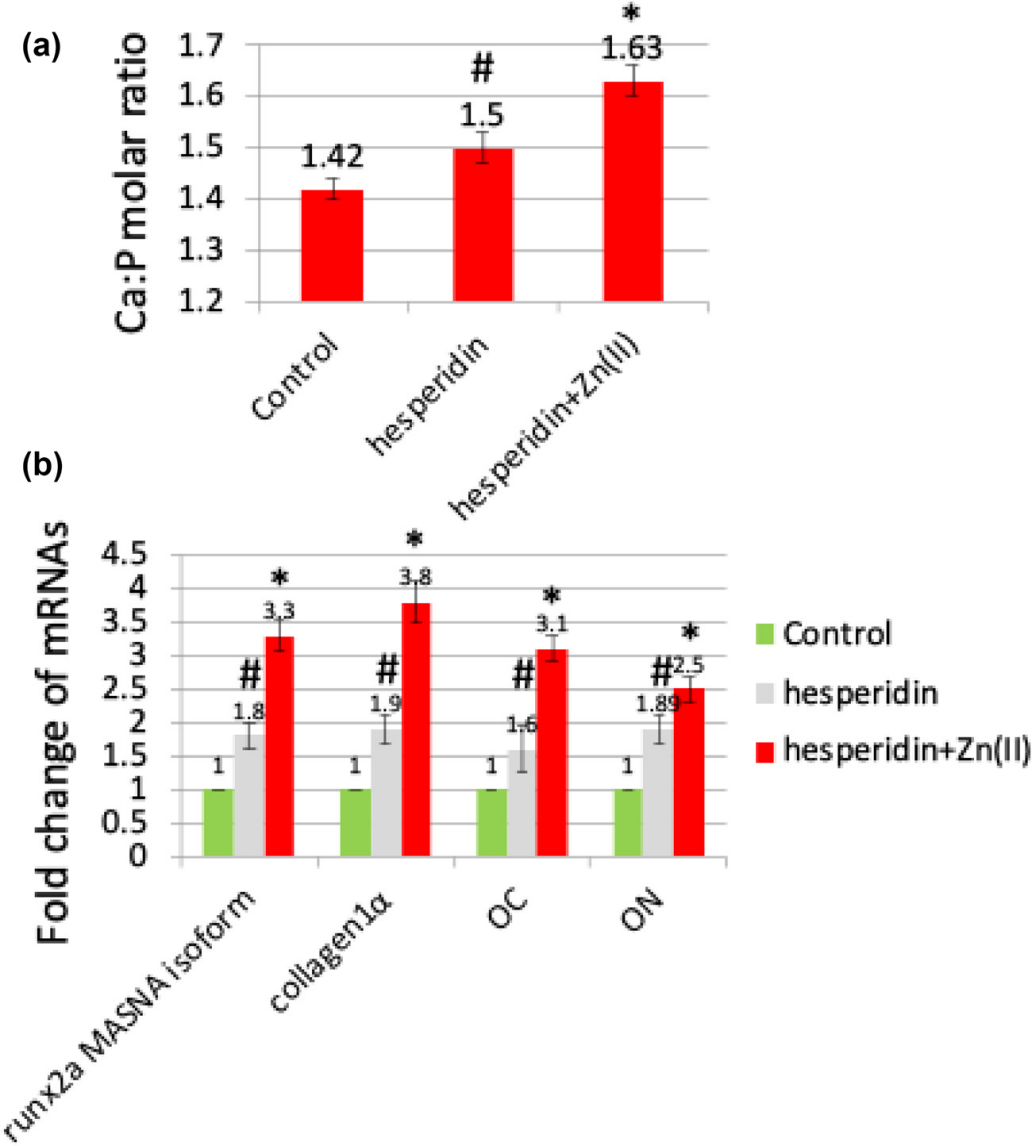
After 14 days of treatment, mineral analysis of the scales was conducted using ICP-MS, and the calcium-to-phosphorus (Ca:P) molar ratio was determined (a). The mRNA expression levels of osteoblast marker genes, including runx2a MASNA isoform, collagen1α, OC, and ON, were analyzed by real-time RT-PCR (b). * denotes a significant increase compared to the control group, while # indicates a significant increase compared to hesperidin treatment.

During our study on the impact of hesperidin and its zinc(ii) complex on bone formation, we administered hesperidin and hesperidin–Zn(ii) complex (at a concentration of 10 μg/mL) to adult zebrafish for 14 days in a controlled laboratory environment. After the treatment, the scales were removed and analyzed using several methods to evaluate calcium deposition, mineral content, and the expression of osteoblast markers. The unique qualities of zebrafish scales, such as their clarity, lack of epidermal coating, and ability to regenerate, make them an excellent model for researching osteoblast characteristics. The initial von Kossa (Figure 7a) and Alizarin red (Figure 7b) staining showed higher levels of calcium deposition in scales treated with hesperidin compared to the control. Additionally, scales treated with the hesperidin–Zn(ii) complex exhibited even greater augmentation of calcium deposition quantification (Figure 7c and d). ICP-MS analysis revealed increased concentrations of calcium, phosphorus, and magnesium in scales treated with hesperidin. Notably, scales treated with hesperidin–Zn(ii) complex showed a significant boost in these mineral levels (Figure 8a). The calculation of the calcium/phosphorus molar ratio provided additional confirmation of the stimulating effect of hesperidin and its zinc(ii) complex on mineralization. A considerable increase was detected in comparison to the control. In addition, examination of the expression of genes that indicate the presence of osteoblasts, such as runx2a MASNA isoform, collagen 1α, OC, and ON, showed an increase in hesperidin-treated scales compared to the control. Furthermore, there was a greater increase in scales treated with the hesperidin–Zn(ii) complex (Figure 8b). The current study aligns with existing literature highlighting the role of hesperidin in osteoblast differentiation and bone formation [21,22,23]. Moreover, our findings are consistent with research indicating that the complexation of metals enhances the activity of flavonoids for bone formation, as documented previously [8]. This suggests that the combination of hesperidin with zinc(ii) in our study may have contributed to its efficacy in promoting osteoblast differentiation and bone formation. These collective insights reinforce the potential of hesperidin and its metal complexes as promising candidates for bone health interventions, warranting further exploration in future studies. Overall, these discoveries emphasize the ability of hesperidin to stimulate the growth of bones. This effect is even stronger when hesperidin is combined with zinc(ii), which suggests that hesperidin has great potential for encouraging the development of bone cells and the production of bones in living organisms.

## 4 Conclusion

Our work concludes that the synthesized hesperidin–Zn(ii) combination shows great promise in enhancing the differentiation of osteoblasts and the creation of bone. By conducting thorough investigations both in laboratory conditions (*in vitro*) and in living organisms (*in vivo*), we have confirmed that the hesperidin–Zn(ii) combination is compatible with MG63 cells and chick embryos. Additionally, we have observed that this complex has the ability to increase ALP activity. At an optimum dose of 10 μg/mL, both hesperidin and its zinc(ii) complex showed significant impacts on osteoblast development in mouse MSCs, both at the cellular and molecular levels. More precisely, hesperidin enhanced the process of calcium deposition, increased the activity of ALP, and promoted the expression of important osteoblast markers like Runx2 and type 1 collagen. In addition, the hesperidin–Zn(ii) combination exhibited a higher level of effectiveness in promoting osteoblast development when compared to hesperidin alone. The mechanism of action of the hesperidin–Zn(ii) combination involves the activation of the miR-143-3p/HDAC7 signaling pathway, resulting in elevated miR-143 expression and reduced HDAC7 levels. Furthermore, our results from the zebrafish scale model provide evidence for the ability of hesperidin and its complex to promote bone formation. This is demonstrated by an increase in calcium deposition, a higher Ca:P molar ratio, and an upregulation of genes associated with osteoblasts. Our findings indicate that the hesperidin–Zn(ii) complex has potential as an exceptional biomaterial for bone regeneration compared to hesperidin alone. This presents new opportunities for advancing treatment strategies focused on enhancing bone health and facilitating repair.

## Supplementary Material

Supplementary Table
