# Supplementary material for: Hesperidin and its zinc(ii) complex enhance osteoblast differentiation and bone formation: In vitro and in vivo evaluations
Source: Open Life Sci. 2025 Jun 17;20(1):20221032. doi: 10.1515/biol-2022-1032 (PMC12198943; doi:10.1515/biol-2022-1032)
Supplement: Supplementary Table [file biol-2022-1032-sm.pdf]

Supplementary material

Table S1: Primer sequences used for real time RT-PCR analysis

Gene		5′to3′ Sequence
Runx2	Forward	CAGTTCCCAAGCATTTCATC
	Reverse	TCAATATGGTCGCCAAACAG
Type-1 collagen	Forward	TAACCCCCTCCCCAGCCACAAA
	Reverse	TTCCTCTTGGCCGTGCGTCA
GAPDH	Forward	TTGATGTCATCATACTTGGCAGGT
	Reverse	CAG TCAAGGCTGAGAATGGGA
mir-143	Forward	CAGTGCTGCATCTCTGGTCA
	Reverse	TGCAGAACAACTTCTCTCTTCCT
U6	Forward	CTCGCTTCGGCAGCACA
	Reverse	AACGCTTCACGAATTTGCGT
runx2a masns-	Forward	CTCCCGCTTTAGGACTTCGA
isoform	Reverse	GGAGTCACCGAGCTGAAAAGACT
collagen 1α2	Forward	GGAAACCTGAAGAAGGCTGTGT
	Reverse	TGAAAGTGAAGCGGCTGTTG
osteocalcin	Forward	TGGCCTCTATCATCATGAGACAGA
	Reverse	CTCTCGAGCTGAAATGGAGTCA
osteopontin	Forward	CGCTCAGCAAGCAGTTCAGA
	Reverse	AGAATAGGAGGTGGCCGTTGA
β-actin	Forward	CAACAGGGAAAAGATGACACAGAT
	Reverse	CAGCCTGGATGGCAACGT
